# Regulated cell death in sepsis-associated liver injury: molecular mechanisms and therapeutic implications

**DOI:** 10.3389/fimmu.2026.1740461

**Published:** 2026-02-11

**Authors:** Kun Zou, Na Wang, Linyu Wang, Zhongqiang Zhu, Mengyuan Gu, Junqiang Zhang

**Affiliations:** 1 Department of General Surgery, The Second Hospital and Clinical Medical School, Lanzhou University, Lanzhou, Gansu, China; 2 Department of Critical Care Medicine, The Second Hospital and Clinical Medical School, Lanzhou University, Lanzhou, Gansu, China

**Keywords:** bile acid metabolism, ferroptosis, immune dysregulation, lipid peroxidation, pyroptosis, regulated cell death, sepsis-associated liver injury

## Abstract

Sepsis-associated liver injury (SALI) is a critical determinant of sepsis prognosis, characterized by extensive hepatocellular death and dysregulated immune responses. Emerging evidence highlights the pivotal role of regulated cell death (RCD) — including apoptosis, necroptosis, pyroptosis, and ferroptosis — in driving hepatic dysfunction and systemic inflammation. These cell death modalities, once considered distinct, are now recognized as components of an interconnected network that integrates inflammatory, metabolic, and oxidative signals within the liver’s unique immunometabolic microenvironment. This review systematically summarizes the molecular mechanisms of major RCD pathways implicated in SALI, and elucidates their crosstalk and convergence through shared mediators such as caspase-8, the NLRP3 inflammasome, lipid peroxidation, and liver-specific metabolic regulators including bile acid signaling. We further discuss key signaling cascades including PI3K/Akt, Nrf2, and NF-κB that orchestrate RCD execution and inflammatory amplification in SALI. By integrating mechanistic insights with emerging translational perspectives, this review highlights RCD as a unifying framework for understanding liver injury and identifying therapeutic entry points to restore hepatic and systemic homeostasis during sepsis.

## 1 Introduction

Sepsis, defined as a dysregulated host response to infection causing life-threatening organ dysfunction, remains a major global public health burden (1). In 2017, an estimated 48.9 million sepsis cases and 11 million associated deaths occurred worldwide, accounting for nearly 20% of global mortality (2). Among the affected organs, the liver serves as both a key target and an active participant in sepsis pathophysiology. Sepsis-associated liver injury (SALI) occurs in approximately one-third of patients and carries a mortality rate between 54%–68% (3, 4). Hepatic dysfunction has thus been recognized as an independent predictor of poor prognosis in sepsis.

As a central metabolic and immunological hub, the liver plays vital roles in pathogen clearance, cytokine production, and acute-phase protein synthesis. However, this centrality also renders it highly susceptible to damage under excessive systemic inflammation and oxidative stress (5, 6). Recently, regulated cell death (RCD) has emerged as a critical contributor to hepatocellular injury during sepsis. Distinct forms of RCD—including apoptosis, necroptosis, pyroptosis, and ferroptosis—cooperate and intersect to drive hepatocyte loss and immune dysregulation (7–9). Understanding their mechanisms and interconnections is essential for developing targeted interventions. This review summarizes the current knowledge of these major RCD pathways in SALI, highlights their molecular crosstalk, and discusses key intracellular signaling cascades that regulate these processes.

## 2 Major forms of RCD in SALI

### 2.1 Apoptosis: classical caspase-mediated hepatocyte death

**Figure 1:**
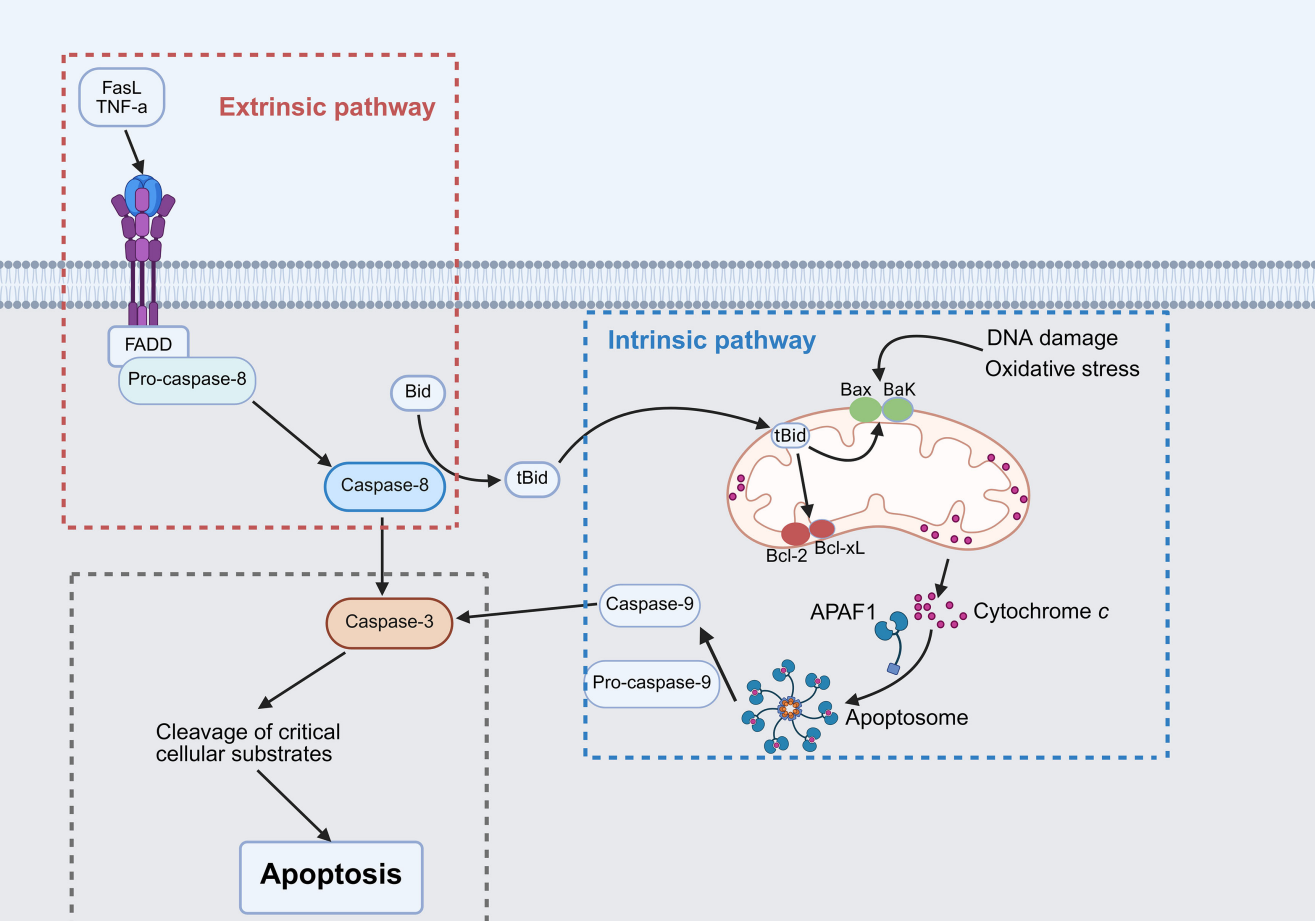
Schematic representation of apoptotic pathways. The intrinsic pathway is initiated by intracellular stress signals, while the extrinsic pathway is triggered by extracellular death ligands. Both pathways converge on activated caspase-3, which cleaves cellular substrates to induce apoptosis.

Apoptosis is a caspase-dependent, non-inflammatory form of RCD that eliminates damaged or infected cells to maintain homeostasis (10). Morphologically, apoptotic cells exhibit membrane blebbing, chromatin condensation, and formation of apoptotic bodies. Apoptosis proceeds through two canonical pathways: intrinsic and extrinsic (**Figure 1**). The intrinsic (mitochondrial) pathway is regulated by Bcl-2 family proteins that control mitochondrial membrane permeability (11). Activation of Bax/Bak promotes cytochrome *c* (Cyt-c) release, triggering apoptosome formation and sequential activation of caspase-9 and caspase-3 (12). The extrinsic (death receptor) pathway is initiated when extracellular ligands such as tumor necrosis factor (TNF) or Fas ligand (FasL) bind their respective receptors, leading to Fas-associated protein with a death domain (FADD) recruitment and caspase-8 activation, which directly cleaves caspase-3 (13).

In SALI, lipopolysaccharide (LPS) and inflammatory cytokines promote hepatocyte apoptosis through caspase-3 activation and dysregulation of Bax/Bcl-2 expression (14, 15). Bid serves as a molecular bridge between intrinsic and extrinsic pathways; its deletion attenuates hepatocyte apoptosis and improves survival in septic models (16). Upstream regulators, including endoplasmic reticulum stress (17), P2Y2 purinergic signaling (18), and microRNAs (e.g., miR-30a, miR-103a-3p) (19, 20), further fine-tune apoptotic cascades. Therapeutically, anti-apoptotic agents including menthol (21), obeticholic acid (22), and fibroblast growth factor 19 (FGF19) (23) confer hepatoprotection by suppressing caspase activation and oxidative stress, with menthol additionally improving survival, and obeticholic acid and FGF19 restoring bile acid homeostasis in experimental sepsis models. Collectively, apoptosis represents a central mechanism of hepatocyte loss in SALI, and modulation of its key regulators offers promising therapeutic potential.

### 2.2 Necroptosis: programmed necrosis amplifying inflammation

**Figure 2:**
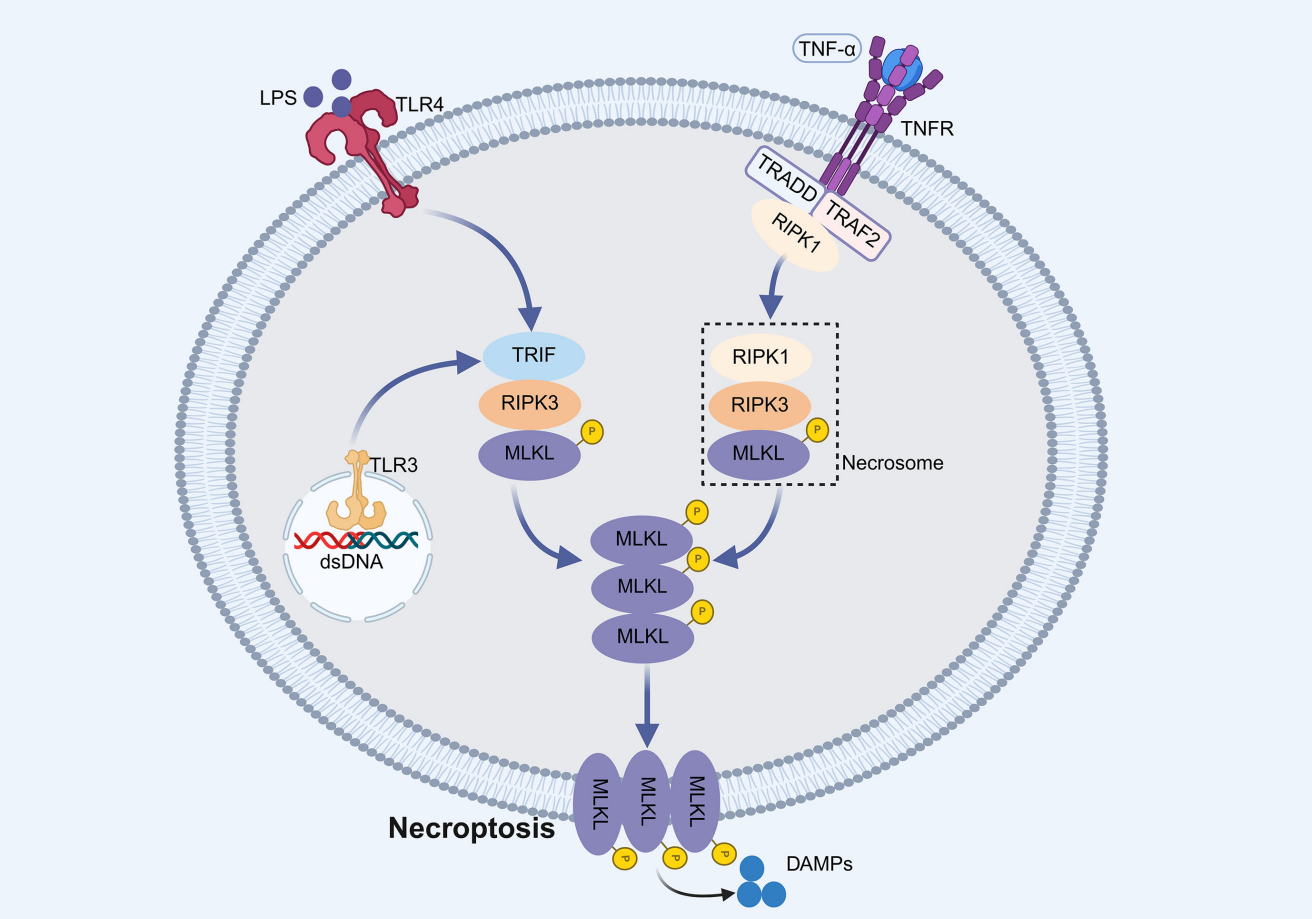
Schematic representation of necroptosis pathways. Activation of death receptors or Toll-like receptors recruits RHIM domain-containing proteins to engage RIPK3. Activated RIPK3 phosphorylates MLKL, which oligomerizes and translocates to the plasma membrane, resulting in plasma membrane rupture and necroptotic cell death.

Necroptosis is a caspase-independent, regulated necrotic process characterized by cell swelling, plasma membrane rupture and release of damage-associated molecular patterns (DAMPs) (24). It is primarily governed by receptor-interacting protein kinase 1 (RIPK1)–RIPK3–mixed lineage kinase domain-like protein (MLKL) signaling axis (25). Upon activation of death receptors (e.g., TNFR) or certain Toll-like receptors (TLRs), RIPK1 recruits RIPK3 through RIP homotypic interaction motif (RHIM)-mediated interactions; RIPK3 then phosphorylates MLKL, which oligomerizes and disrupts the plasma membrane (26, 27) (**Figure 2**).

Necroptosis is markedly activated in septic livers, evidenced by elevated RIPK1, RIPK3, and p-MLKL expression (28–30). While moderate necroptosis may contribute to antimicrobial defense (31), excessive activation amplifies inflammation and tissue injury (30). Intriguingly, pharmacological inhibition of necroptosis (e.g., necrostatin-1) has yielded inconsistent outcomes—attenuating necroptosis but sometimes worsening survival—highlighting its context-dependent dual role in SALI (32). Thus, selective modulation rather than complete suppression may be necessary for therapeutic efficacy.

### 2.3 Pyroptosis: inflammasome-driven inflammatory cell death

**Figure 3:**
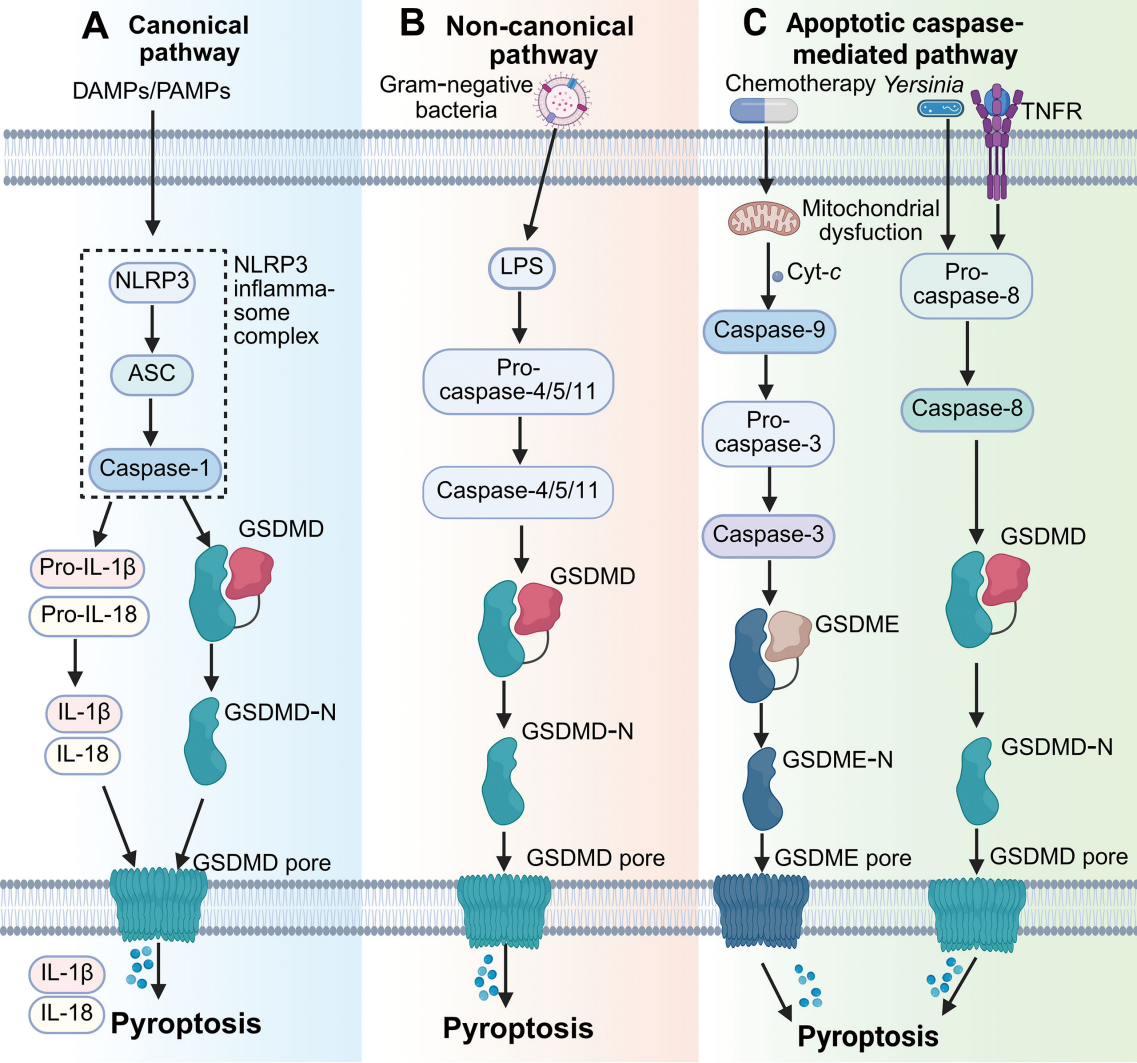
Schematic representation of the major pyroptosis pathways. **(A)** In the canonical pathway, DAMPs or PAMPs activate inflammasomes, leading to caspase-1 activation, which cleaves GSDMD to form membrane pores and facilitates the release of IL-1β and IL-18. **(B)** In the non-canonical pathway, intracellular LPS from Gram-negative bacteria directly activates caspase-4/5/11, resulting in GSDMD cleavage and pyroptosis. **(C)** In the apoptotic caspase-mediated pathway, caspase-3 cleaves GSDME, while caspase-8 cleaves GSDMD, both contributing to pyroptotic execution.

Pyroptosis is an inflammatory form of RCD mediated by gasdermin family proteins and inflammatory caspases (33). Pyroptosis can be initiated via three major routes: the canonical inflammasome pathway, the non-canonical inflammasome pathway, and the caspase-3/gasdermin E (GSDME)-mediated pathway. In the canonical pathway, activation of pattern recognition receptors (PRRs), such as nucleotide-binding domain and leucine-rich repeat related (NLR) family pyrin domain containing 3 (NLRP3), recruits apoptosis-associated speck-like protein containing a caspase-recruitment domain (ASC) and caspase-1 to assemble the inflammasome complex. Activated caspase-1 then cleaves gasdermin D (GSDMD) and facilitates the release of IL-1β and IL-18 (34, 35). The non-canonical pathway is triggered by cytosolic LPS directly activating caspase-4/5/11, leading to GSDMD cleavage and pyroptosis independently of NLRP3 (36). Additionally, pyroptosis can be engaged through a caspase-3/GSDME-dependent pathway, in which apoptotic caspase-3 cleaves GSDME, thereby converting apoptosis into secondary pyroptotic cell death (37). Under certain infectious conditions, caspase-8 can also induce pyroptosis via GSDMD processing, such as during *Yersinia* infection (38) (**Figure 3**).

In SALI, excessive pyroptosis amplifies hepatic inflammation. LPS and DAMPs induce caspase-11–GSDMD–dependent hepatocyte pyroptosis, while GSDME-mediated pyroptosis further aggravates damage (9, 39). Inflammasome activation in Kupffer and hepatocytes disrupts immune tolerance and promotes cytokine storm and hepatic necroinflammation (40, 41). Therapeutic agents such as maresin 1, samotolisib, and irisin have shown efficacy in inhibiting NLRP3 inflammasome or caspase-11–mediated pyroptosis (42–44). Hence, controlled modulation of pyroptosis represents a promising avenue to mitigate inflammatory liver injury and restore immune balance during SALI.

Importantly, beyond serving as an executioner of inflammatory cell death, pyroptosis critically shapes immune homeostasis during sepsis (45). Excessive inflammasome-driven pyroptosis results in robust release of IL-1β and IL-18, enhancing pathological leukocyte recruitment, and aggravating tissue inflammation. In parallel, sustained pyroptotic death of innate immune cells, including macrophages and dendritic cells, contributes to immune cell depletion and subsequent immunosuppression (46). Therefore, therapeutic modulation of pyroptosis has the potential to rebalance host immunity during sepsis by simultaneously restraining hyperinflammation in the early phase and preserving immune cell competence in later stages, thereby limiting immunopathology while preventing immune paralysis (47). This dual role positions pyroptosis as a critical regulatory node linking inflammatory amplification to immune dysregulation in SALI.

### 2.4 Ferroptosis: iron-dependent lipid peroxidation–induced hepatocyte injury

**Figure 4:**
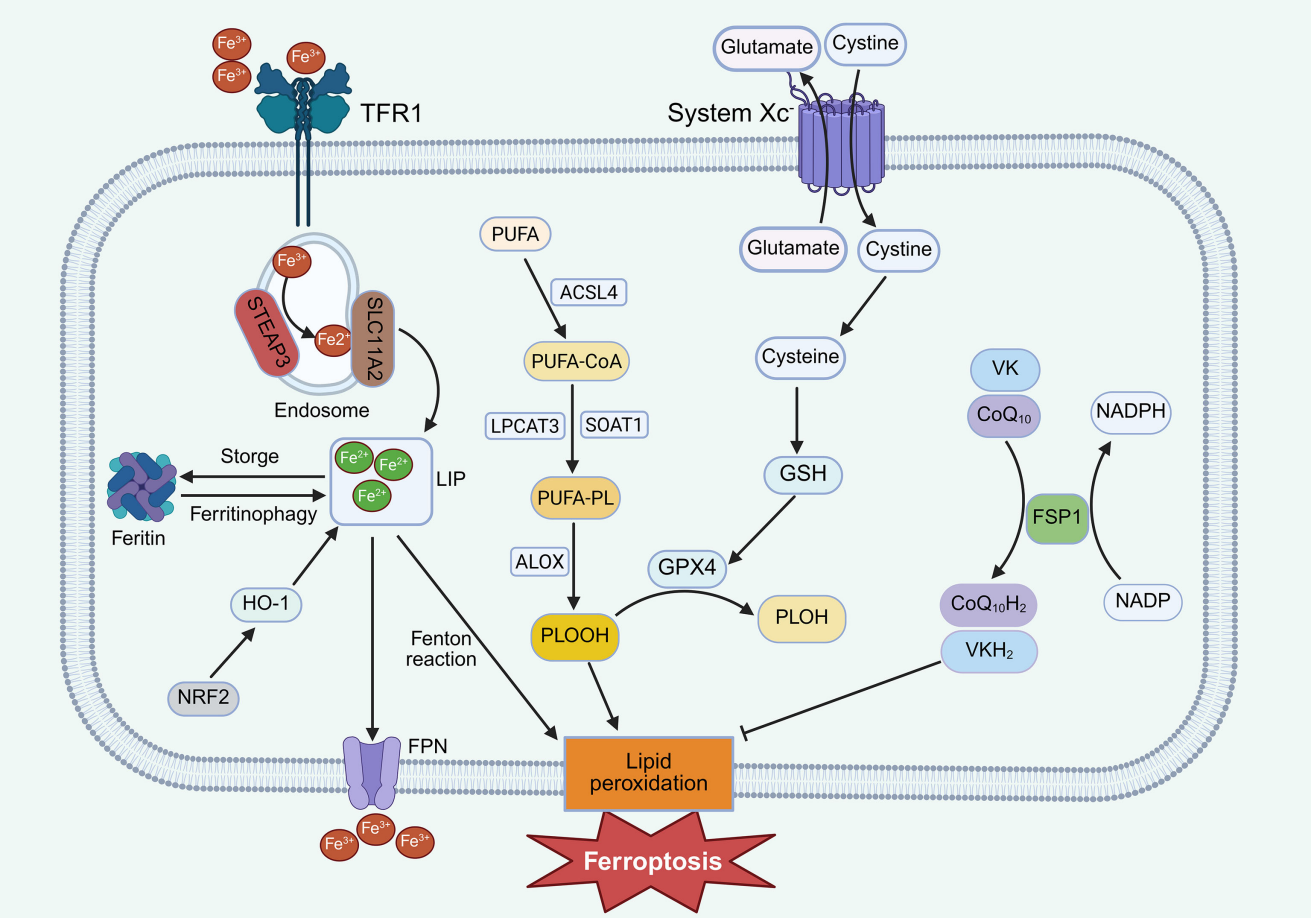
Schematic representation of ferroptosis pathways. Extracellular Fe³^+^ enters cells via TFR1-mediated endocytosis and is reduced to Fe²^+^ in the labile iron pool (LIP). Excess Fe²^+^ catalyzes Fenton reactions, producing ROS that induce oxidative membrane damage. The system Xc^-^ transporter imports cystine to sustain GSH synthesis, while GPX4 utilizes GSH to convert PLOOHs to non-toxic PLOHs, thereby suppressing ferroptosis. In addition, FSP1-mediated regeneration of reduced CoQ_10_H_2_and VKH_2_ provides an independent antioxidant system that scavenges lipid radicals and suppresses ferroptosis.

Ferroptosis, first described by Dixon et al. in 2012, is driven by iron-dependent lipid peroxidation resulting from redox imbalance (48). It features mitochondrial shrinkage, cristae loss, and outer membrane rupture (49). Mechanistically, excess Fe²^+^ catalyzes the Fenton reaction, generating reactive oxygen species (ROS) that oxidize polyunsaturated fatty acids (PUFAs)-containing phospholipids (50, 51). Two antioxidant systems counteract this process: the cystine/glutamate antiporter (system Xc^-^)–glutathione (GSH)–glutathione peroxidase 4 (GPX4) axis, converting toxic phospholipid hydroperoxides to non-toxic forms (52); the ferroptosis suppressor protein 1 (FSP1)–coenzyme Q_10_ (CoQ_10_) and vitamin K reduction systems, which scavenge free radicals (53, 54) (**Figure 4**).

Clinically, these molecular features of ferroptosis are highly relevant to sepsis pathophysiology. Red blood cell lysis and hemolysis are common features of severe sepsis and septic shock, resulting in the release of free heme and iron into the circulation (55). Elevated plasma free heme levels have been consistently associated with disease severity, organ dysfunction, and increased mortality in septic patients (56, 57). Once released, heme is rapidly taken up by the liver, where its degradation and the ensuing iron overload promote oxidative stress and lipid peroxidation. This heme-driven iron excess provides a direct pathological link between systemic hemolysis during sepsis and ferroptosis-prone hepatocyte injury, thereby connecting clinical sepsis phenotypes with iron-dependent lipid peroxidation and RCD in SALI (58).

In SALI, inhibition of ferroptosis mitigates hepatic dysfunction and improves survival. Key regulators include G protein-coupled receptor 116 (GPR116), which promotes ferroptosis by suppressing GPX4 (59), and Yes-associated protein 1 (YAP1), which prevents ferritinophagy (60). Protective factors such as milk fat globule-EGF factor 8 (MFG-E8) and irisin enhance GPX4 expression and antioxidant defenses (61, 62). Other mediators, including pentraxin-3, neuregulin 4, and maresin 1, also protect against SALI by modulating ferroptosis-related signaling pathways such as the nuclear factor erythroid 2- related factor 2 (Nrf2)/solute carrier family 7 member 11 (SLC7A11)/GPX4 axis (63–65). Pharmacological inhibition, for instance by quercetin, suppresses ferritinophagy and reduces intracellular iron overload, thereby mitigating LPS-induced injury (66). Collectively, ferroptosis represents a critical intersection between oxidative stress, lipid metabolism, and hepatocyte death in sepsis.

### 2.5 Comparative features of major RCD modalities in SALI

Although apoptosis, pyroptosis, necroptosis, and ferroptosis represent distinct forms of RCD, they contribute to SALI through partially overlapping yet functionally divergent mechanisms. Apoptosis predominates in hepatocytes during early phases of sepsis and is generally considered less immunogenic, serving as a homeostatic mechanism to eliminate damaged cells. In contrast, pyroptosis and necroptosis are highly inflammatory and occur predominantly in innate immune cells such as Kupffer cells and infiltrating macrophages, amplifying cytokine release and hepatic immune dysregulation. Ferroptosis, driven by iron-dependent lipid peroxidation, uniquely links metabolic vulnerability with oxidative stress and is particularly relevant to hepatocytes given the liver’s central role in iron handling and lipid metabolism.

Importantly, these RCD modalities are not mutually exclusive. Their temporal activation, cellular distribution, and inflammatory potential collectively determine whether the hepatic response to sepsis favors adaptive injury resolution or progresses toward uncontrolled inflammation, immune dysfunction, and multi-organ failure. Recognizing both the shared and distinct features of these death programs provides a clearer mechanistic framework for understanding SALI and for identifying stage- and cell-specific therapeutic targets.

## 3 Crosstalk and convergence among RCD pathways in SALI

While apoptosis, necroptosis, pyroptosis, and ferroptosis have traditionally been investigated as independent RCD programs, accumulating evidence indicates that these pathways rarely operate in isolation during SALI. Importantly, the liver represents a uniquely vulnerable organ in which intensive metabolic activity, abundant mitochondria, iron-rich physiology, and dense innate immune surveillance converge. Within this specialized microenvironment, hepatocytes and Kupffer cells engage distinct but interconnected RCD programs in response to septic insults. Rather than functioning as independent execution mechanisms, multiple RCD modalities are often co-activated simultaneously and engage in extensive molecular crosstalk, leading to signal amplification and pathway convergence that shape inflammatory liver injury. This section therefore focuses on the interconnected nature of RCD pathways in SALI, highlighting shared molecular nodes and cooperative mechanisms within the hepatic context.

### 3.1 Caspase-8: a molecular switch linking apoptosis, necroptosis, and pyroptosis

Caspase-8 serves as a central switch coordinating multiple RCD modalities. Under physiological conditions, it activates caspase-3/7 to initiate apoptosis while simultaneously cleaving RIPK1 and RIPK3 to suppress necroptosis. In the septic microenvironment, increased cFLIP expression restrains caspase-8 activation within death receptor complexes, suppressing apoptotic execution and reshaping downstream signaling. Under conditions of sustained caspase-8 inhibition, this shift may permit RIPK1–RIPK3 complex formation and increase susceptibility to necroptotic or other inflammatory cell death programs (67). When both apoptosis and necroptosis are blocked, caspase-8 serves as a scaffold that interacts with the ASC to activate NLRP3-dependent pyroptosis (68). Moreover, under specific stimuli such as *Yersinia* infection, caspase-8 directly cleaves GSDMD to trigger pyroptosis independent of caspase-1 (69).

### 3.2 NLRP3 inflammasome: a convergence hub of inflammatory RCD

The NLRP3 inflammasome acts as a key integrator of inflammatory cell death and plays an important role in SALI. Classically, it activates caspase-1 to cleave GSDMD in immune cells, leading to IL-1β and IL-18 release during pyroptosis. Beyond this canonical role, NLRP3 interacts with other death pathways. During apoptosis, NLRP3 colocalizes with ASC and caspase-8 in mitochondria-associated speck-like complexes (70). In the absence of caspase-1, NLRP3 inflammasomes directly engage caspase-8 to initiate apoptosis and cytokine processing (71). Iron overload promotes NLRP3 activation through the cyclic GMP-AMP synthase (cGAS)-stimulator of interferon gene (STING) pathway, while ROS generated by NLRP3 activation exacerbate lipid peroxidation, thereby linking inflammasome signaling to ferroptosis (72). Ferroptosis in Kupffer cells can contribute to liver injury by amplifying oxidative and inflammatory signals. Moreover, activation of the NLRP3 inflammasome in Kupffer cells directly contributes to hepatocyte death, as demonstrated in a Kupffer cell–hepatocyte co-culture model in which Kupffer cells isolated from *Nlrp3*-deficient mice induced significantly less hepatocyte death than their wild-type counterparts (73). Moreover, MLKL, a key effector of necroptosis, can also activate NLRP3 in macrophages, amplifying inflammation and forming a feedback loop between necroptosis and pyroptosis (74). These findings suggest that NLRP3 represents a pivotal node integrating inflammatory and death signals in SALI.

### 3.3 Lipid peroxidation: a shared effector connecting oxidative and inflammatory cell death

Lipid peroxidation represents a critical biochemical process that integrates oxidative stress with multiple RCD pathways in SALI. In polymicrobial sepsis models, hepatic lipid peroxidation is significantly increased and parallels enhanced hepatocyte apoptosis, implicating lipid peroxidation as a key contributor to sepsis-induced hepatocyte injury (75). Under septic stress, excessive ROS production promotes peroxidative damage to mitochondrial membrane, thereby facilitating Cyt-c release and initiating mitochondria-dependent apoptosis in hepatocytes (76). Beyond apoptosis, lipid peroxidation intersects with inflammatory cell death pathways. During sepsis, lipid peroxidation drives pyroptosis through caspase-11-mediated GSDMD cleavage and phospholipase C gamma 1-dependent activation of GSDMD fragments (77). Notably, lipid peroxidation product can exert context-dependent regulatory effects on inflammasome signaling: 4-hydroxynonenal (4-HNE) inhibits NLRP3 inflammasome activation and macrophage pyroptosis by impairing NEK7-NLRP3 interactions (78), whereas the PUFA -derived lipid mediator resolving D1 suppresses caspase-1/GSDMD-dependent pyroptosis in the liver of septic mice (79). Ferroptosis, intrinsically characterized by iron-dependent lipid peroxidation, is particularly relevant to SALI given the liver’s central role in iron storage and lipid metabolism (80). This process is counteracted by GPX4-dependent and -independent antioxidant defenses. Collectively, these findings highlight lipid peroxidation not merely as a downstream consequence of oxidative stress, but as a shared effector that both drives and fine-tunes the crosstalk among apoptotic, pyroptotic, and ferroptotic pathways in SALI.

From a translational perspective, recognizing lipid peroxidation as both a trigger and a modulator of RCD in SALI has important therapeutic implications. Antioxidant approaches that curb ROS-driven lipid peroxidation, iron chelation therapies that limit the labile iron pool fueling ferroptosis, and interventions enhancing GPX4-dependent or GPX4-independent lipid peroxide detoxification may collectively restrain systemic inflammatory amplification during sepsis. By reducing the release of DAMPs and pro-inflammatory lipid species from injured hepatocytes and hepatic macrophages, such strategies could plausibly attenuate cytokine storm propagation, preserve immune cell function, and mitigate downstream multi-organ injury. Therefore, modulation of lipid peroxidation emerges as a unifying therapeutic entry point linking hepatic protection with systemic immune homeostasis in sepsis.

### 3.4 Bile acid metabolism: a liver-specific modulator of RCD in sepsis

Bile acids are liver-derived metabolites that play essential roles in lipid digestion, metabolic homeostasis, and immune regulation. During sepsis, bile acid metabolism is profoundly disrupted and is increasingly recognized as a contributor to SALI (81). Excessive accumulation of bile acids has been shown to exert direct cytotoxic effects on hepatocytes and to activate inflammatory signaling cascades, thereby promoting multiple forms of cell death, including apoptosis, necrotic cell death, and pyroptosis (82). Experimental endotoxemia and polymicrobial sepsis models consistently demonstrate marked alterations in circulating and hepatic bile acid profiles, accompanied by cholestasis and hepatic inflammation. Notably, pharmacological activation of the bile acid receptor farnesoid X receptor (FXR) partially restores bile acid homeostasis and alleviates liver injury, underscoring the regulatory importance of bile acid signaling in SALI (83). Mechanistically, bile acids can function as DAMPs capable of activating NLRP3 inflammasome in macrophages, whereas FXR signaling negatively regulates NLRP3 activity and downstream inflammatory cytokine production (84). In addition to FXR, the bile acid receptor Takeda G protein–coupled receptor 5 (TGR5) represents a critical liver-enriched immunometabolic sensor during sepsis. Recent evidence demonstrates that TGR5 expression is significantly upregulated in the liver and macrophages following bacterial infection and LPS challenge, indicating an adaptive hepatic response to septic stress (85). Activation of TGR5 not only drives the polarization of hepatic macrophages toward an anti-inflammatory phenotype but also suppresses the activation of the NLRP3 inflammasome, thereby mitigating hepatocyte pyroptosis and inflammatory damage (86). Clinically, patients with septic shock exhibit markedly elevated circulating bile acids levels, particularly in the presence of hepatic dysfunction. These alterations have been shown to be independently associated with increased short-term mortality and may serve as early biomarkers for risk stratification in critically ill patients (87). Given the liver’s central role in bile acid synthesis, enterohepatic circulation, and bile acid receptor–mediated immunometabolic regulation, dysregulated bile acid metabolism is likely to exacerbate oxidative stress, mitochondrial dysfunction, inflammatory signaling, and RCD pathways in both hepatocytes and hepatic macrophages during SALI. Collectively, these findings establish bile acid metabolism as a liver-specific metabolic axis linking immunometabolic disturbance to inflammatory RCD during sepsis.

## 4 Signaling pathways orchestrating RCD and inflammation in SALI

While the preceding sections detailed the molecular mechanisms and crosstalk among distinct forms of RCD, these processes do not occur autonomously. Instead, the initiation and intensity of RCD are tightly governed by upstream signaling pathways that integrate inflammatory, oxidative, and metabolic disturbances during sepsis. Therefore, understanding how key intracellular signaling cascades orchestrate RCD and inflammation is essential for elucidating the pathogenesis of SALI.

Among the numerous signaling pathways implicated in SALI, phosphoinositide 3-kinase/protein kinase B (PI3K/Akt), Nrf2, and nuclear factor κ-B (NF-κB) were selected for focused discussion based on three considerations. First, these pathways represent distinct but complementary regulatory axes governing cell survival, oxidative stress responses, and inflammatory signaling—three central determinants of RCD fate in SALI. Second, accumulating evidence indicates that each of these pathways directly intersects with multiple RCD modalities, including apoptosis, pyroptosis, necroptosis, and ferroptosis, rather than regulating a single death program. Third, these signaling cascades are highly druggable and have been repeatedly validated in experimental SALI models, highlighting their translational relevance.

### 4.1 PI3K/Akt pathway: a cytoprotective modulator of hepatocyte survival

The PI3K/Akt pathway is a central signaling cascade regulating cell growth, metabolism, and survival (88). In sepsis, it exerts protective effects by limiting excessive inflammatory cytokine release and enhancing stress adaptation. Inhibition of PI3K aggravates polymicrobial sepsis, whereas its activation mitigates sepsis-related morbidity and mortality (89).

In the context of SALI, PI3K/Akt activation protects hepatocytes from Fas- or TNF-α-induced apoptosis (90). Upstream regulators such as fibroblast growth factor 5 and thymic stromal lymphopoietin attenuate LPS-induced liver injury via PI3K/Akt-dependent anti-apoptotic and autophagic mechanisms (91, 92). Similarly, the long non-coding RNA LncRNA 220 modulates autophagy and apoptosis in Kupffer cells through the miR-5101/PI3K/Akt/mTOR axis (93).

Natural compounds including wedelolactone, aloe-emodin, and curcumin modulate PI3K/Akt signaling to suppress oxidative stress, ferroptosis, apoptosis, and inflammation (94–96). Interestingly, time-restricted feeding alleviates septic liver injury by remodeling gut microbiota, enriching *Lactobacillus murinus*, and activating PI3K/Akt/mTOR signaling to inhibit hepatocyte ferroptosis (97). Collectively, these findings establish the PI3K/Akt pathway as a crucial cytoprotective axis and an attractive therapeutic target for SALI.

### 4.2 Nrf2 pathway: master regulator of antioxidant and anti-ferroptotic defense

Nrf2 is a master transcriptional regulator of cellular antioxidant defense and redox balance (98). Under basal conditions, it is sequestered in the cytoplasm by Kelch-like ECH-associated protein 1 (Keap1); oxidative stress promotes Nrf2 dissociation and nuclear translocation, where it binds antioxidant response elements (AREs) to induce cytoprotective genes (99).

During sepsis, Nrf2 activation suppresses excessive inflammation and improves host survival (100). In SALI, Nrf2 mitigates hepatic injury by repressing oxidative stress and inflammation, while Nrf2 deficiency aggravates liver injury (101). Mechanistically, Nrf2 inhibits ferroptosis through upregulation of SLC7A11, GPX4, and autophagy-related proteins (102). Pharmacological activators such as artemisitene and nobiletin alleviate SALI via Nrf2-mediated antioxidant and anti-ferroptotic signaling (103, 104). Furthermore, Nrf2 activation limits pyroptosis by suppressing NLRP3 inflammasome formation (42, 105, 106). Collectively, Nrf2 acts as a multifaceted guardian integrating antioxidant, anti-ferroptotic, and anti-pyroptotic responses in SALI.

### 4.3 NF-κB signaling pathway: a central driver of inflammation and cell death

NF-κB is a central transcription factor regulating inflammation, immunity, and cell fate decisions (107). Under resting conditions, NF-κB is retained in the cytoplasm by Inhibitor of κB (IκB) proteins. Upon exposure to stimuli such as cytokines, LPS, or oxidative stress, IκB is phosphorylated and degraded, enabling NF-κB translocation to the nucleus, where it binds to target gene promoters and initiates transcription of inflammatory mediators (108).

Excessive activation of NF-κB is a hallmark of sepsis and correlates with increased mortality and poor clinical outcomes (109). Accordingly, inhibition of NF-κB signaling alleviates systemic inflammation and organ dysfunction. In SALI, activation of the TLR4/MyD88/NF-κB axis contributes to LPS-induced hepatocyte apoptosis (110). Golgi phosphoprotein 3 exacerbates endotoxemia-induced liver injury via inhibition of Akt/NF-κB signaling, whereas its knockdown mitigates damage (111). Multiple agents—including neoastilbin, L-ascorbic acid 6-palmitate, rupatadine, and resveratrol—suppress NF-κB to reduce oxidative stress, apoptosis, and inflammation (112–115). Thus, NF-κB represents a pivotal proinflammatory driver linking immune dysregulation and cell death in SALI.

Collectively, these signaling pathways do not operate in isolation. PI3K/Akt increases cellular resilience to apoptotic and ferroptotic stress, Nrf2 sets antioxidant capacity and ferroptotic sensitivity, whereas NF-κB amplifies inflammatory RCD programs such as pyroptosis and necroptosis. The dynamic balance among these pathways critically shapes whether hepatocytes and hepatic immune cells undergo adaptive survival, inflammatory death, or irreversible metabolic collapse during SALI progression. Importantly, emerging liver-targeted drug designs highlight the translational feasibility of modulating these inflammatory signaling pathways in SALI. For example, a camptothecin–bile acid conjugate exhibited pronounced hepatic targeting and markedly attenuated liver injury in septic mice by inhibiting NF-κB signaling (116). This bile acid-based delivery strategy not only enhanced hepatic drug exposure but also reduced systemic toxicity, underscoring the importance of liver-specific targeting and hepatic metabolism in the development of effective therapies for SALI.

## 5 Conclusions and perspectives

SALI represents a multifactorial process driven by interconnected RCD pathways within the liver’s unique immunometabolic environment. Apoptosis, necroptosis, pyroptosis, and ferroptosis form a dynamic and partially overlapping network that amplifies hepatic inflammation and dysfunction in sepsis. Crosstalk mediated by caspase-8, NLRP3 inflammasome, and lipid peroxidation integrates immune, metabolic, and oxidative stress signals. Meanwhile, the PI3K/Akt, Nrf2, and NF-κB pathways orchestrate the balance between cell survival and death.

Future research should focus on (1) delineating temporal and cell-type–specific dynamics of RCD activation during sepsis progression (2); identifying liver-enriched immunometabolic regulators that modulate multiple RCD modalities; and (3) developing combinational therapies targeting shared signaling hubs to achieve hepatic protection while preserving systemic immune homeostasis. A deeper understanding of these networks will pave the way for precision interventions to restore hepatic and systemic immune homeostasis in sepsis.
